# Factors associated with patient payments exceeding National Health Insurance fees and out-of-pocket payments in Lao PDR

**DOI:** 10.1080/16549716.2020.1791411

**Published:** 2020-08-03

**Authors:** Kongmany Chaleunvong, Bounfeng Phoummalaysith, Bouaphat Phonvixay, Manithong Vonglokham, Vanphanom Sychareun, Jo Durham, Dirk Essink

**Affiliations:** a Institute of Research and Education Development, University of Health Sciences, Vientiane, Lao PDR; b Director General of the Lao National Health Insurance, Ministry of Health, Vientiane, Lao PDR; c Vice Director General of the Lao National Health Insurance, Ministry of Health, Vientiane, Lao PDR; d Lao Tropical Institute and Public Health, Lao PDR; e Faculty of Public Health, University of Health Sciences, Vientiane, Lao PDR; f School of Public Health and Social Work, Faculty of Health, Queensland University of Technology, Kelvin Grove QLD, Australia; g Athena Institute, Faculty Science, VU University Amsterdam, Amsterdam, Netherlands

**Keywords:** LEARN: Sexual Reproductive Health, ANC and Nutrition, Health insurance, out-of-pocket payment, user fees, universal health coverage, informal sector

## Abstract

**Background:**

Attaining universal health coverage is a target in the Sustainable Development Goals. In Lao PDR, to achieve universal health coverage, the government is implementing a national insurance scheme, initially targeting the informal sector.

**Objective:**

The purpose was to assess: i) the percentage of NHI patients who paid above the scheduled amount, based on individual billing payment; and ii) the factors related to overpayment.

**Methods:**

Descriptive cross-sectional study based on a structured questionnaire administered at health facilities in face-to-face interviews with 1,850 patients in six provinces.

**Results:**

All 1,850 participants worked in the informal sector. Of these, 78.8% of respondents (77.9% of in-patients; 79.5% of out-patients) made co-payments or were exempted from. Factors associated with in-patients paying above the scheduled fee were living in the province and district (OR = 2.8; 95%CI 1.2 to 6.3); not having documents with them (OR = 21.2; 95%CI 5.6 to 80.3); or not having documents (OR: 7.8; 95% CI 2.1 to 28.6). Significant factors associated with additional costs for out-patients were level of facility used at the provincial hospital (OR:1.4; 95% CI 1.1 to 1.9); older age (OR = 2.2; 95%CI 1.5 to 3.1); living in the province and district (OR = 2.3; 95%CI 1.5 to 3.7); living more than 5 km from the facility (OR = 1.4; 95%CI 1.1 to 1.9); buying medicine or supplies outside of the health facility (OR: 5.6; 95% CI 3.1 to 10.2); not bringing documents (OR:9.1; 95% CI 6.1 to 13.5), not having the right documents (OR: 8.9; 95% CI 5.4 to 14.8).

**Conclusions:**

A number of patients paid above scheduled fee rates, which may deter people from utilising services when needing them. There is a need for increased understanding of the benefits of the national insurance scheme among patients and healthcare staff.

## Background

Universal health coverage is a key health target in the Sustainable Development Goals (SDGs). The World Health Organization (WHO) defines universal health coverage (UHC) as ‘access to key promotive, preventive, curative and rehabilitative health interventions for all at an affordable cost’ [1]. The underlying principle is that individuals should be able to use the health services they need, when they need them, without adverse financial consequences [1,2] Financial protection, therefore, is a key dimension of UHC [1–3]. To achieve UHC, WHO suggests governments should use a risk-pooling, prepayment approach [1]. Options include prepaid private health financing, public insurance or taxes, such as the UK’s National Health Service, employment linked voluntary or mandatory health insurance and government funded insurance for the poor [4]. All these schemes apply the basic principle of risk sharing, but voluntary schemes are often hampered by low enrolment rates and sustainability and can exacerbate inequities in healthcare access [5–7].

In lower-middle income countries (LMICs), most of the workforce is situated in the informal sector and uninsured. Consequently, there is a shift in many LMICs towards National Health Insurance (NHI) schemes with mandatory contributions, pooling at the national level and with government purchasing a package of services for all citizens. These packages usually include a range of services from preventive to acute, long term including and palliative care [2,8]. While some countries with NHI have no point of service fees, others include a fee to moderate demand, where a fraction of the cost of needed health services are paid by the insured.

Lao PDR is an LMIC country that has made consistent and substantial progress against key population health indicators including life expectancy and infant and maternal mortality. Despite improvements, there are high socio-economic and geographic inequalities in coverage of health services and health outcomes, especially related to wealth and ethnicity, as many people of ethnic minority backgrounds live in remote and sparsely populated areas with limited access to services [9]. Health services are mainly public, implemented through a network of health centres, district, provincial, central and specialised hospitals. Each province has one provincial public health hospital and each district has one district hospital and five to ten health centres. Additionally, the military and police sectors provide services for their own cadres and their family. Private pharmacies, clinics and hospitals are also increasingly a part of the healthcare services network. Under a series of reforms, healthcare is financed through a mix of out of pocket payment (OPP), government spending, compulsory health insurance for formal sector workers and voluntary health insurance (community-based health insurance) for the informal sector [9]. Out-of-pocket (OOP) expenditure however remains high. To reduce high OOP expenses and achieve UHC by the year 2025, the government is implementing further financing reforms [9,10]. These reforms include increased government health expenditure as a share of total health expenditure based on GDP, reduced reliance on development assistance for health and merging existing insurance schemes into an overarching NHI scheme [9].

Current health insurance schemes for the formal sector are under the Ministry of Labour and Social Welfare and include the Civil Servant Scheme for the government staff and dependents established in 1995, and subsequently revised in 2006 as the State Authority for Social Security (SASS). The Social Security Organization (SSO) is for workers in the business enterprise sector and their dependents (including public and private enterprises) and was initiated in 2002. These two schemes for workers in the formal sector are compulsory, contributory schemes with the contribution shared fifty-fifty between employers and employees) [11].

Financial protection schemes for the informal sector are under the Ministry of Health and consist of three schemes: government funds for the poor initiated in 2004 (Health Equity Fund), the free delivery and free care for all children under 5 years of age scheme, piloted since 2010 and subsequently rolled out widely, and a voluntary community-based health insurance (CBHI) launched in 2002. However, the implementation of multiple health protection schemes resulted in a fragmented system, with limited coverage and implementation often dependent on donor funding. In addition, there was low enrolment in the voluntary community-based health insurance (CBHI) targeted at the estimated five million people in the informal sector [11]. For informal workers, the combination of low incomes, no social protection and high and often unpredictable OPP can manifest as inequalities in healthcare utilisation and outcomes [12,13].

The Prime Minister’s decree 470 (made in 2012) provided the legal framework for the establishment of the NHI Bureau and the integration of existing social health protection schemes into a single payer system, under the management of the Ministry of Health and NHI Bureau. The National Health Insurance Strategy 2017–2020 outlines the scheme’s objectives, funding flows and operational functions [14]. By integrating the different schemes, the NHI aims to move Lao PDR towards its financial objective of UHC and promote uniformity and improved efficiency, effectiveness and risk-pooling through tax-based funding [14]. The first stage of implementing the NHI scheme has focused those schemes that mainly target the informal sector including the former CBHI scheme, the health equity fund, and free maternal and child healthcare including delivery which have been subsumed under the NHI. The new NHI, covering 17 provinces, was launched in the southern province of Attapeu in August 2016 and subsequently rolled out throughout the country capital, except Vientiane, by the end of 2018. Currently, the coverage of NHI for the informal sector is estimated to be 74% [15], with total health insurance coverage (formal and informal sectors) 94%.

Consistent with the national policy adopted in 2012, expectant mothers and children under 5 years remain exempt from payment, as are people from poor households. Additionally, poor patients receive provisions for transport and other incidental costs. This is important for poor households, especially in rural and remote areas, for which access can be a major barrier to the uptake of healthcare services. Outside of exemptions for maternal and child care and the poor, the NHI scheme requires a small co-payment at the facility level. The co-payment for outpatients at provincial, district hospitals and health centres is 15,000 LAK (1.7$), 10,000 LAK (1.13$) and 5,000 LAK (0.56$) respectively; however, the co-payment for the inpatients at the provincial and district hospital is 30,000 LAK (3.5$) and 5,000 LAK (0.56$) for health centre level. Currently, the NHI package includes a range of services from acute, long term including and palliative care with no limitation on cost but excluding prevention and promotion services, cosmetic and transgender surgery and spectacles. If patients are prescribed medication not available in the facility because of shortages in supply, or because they are not covered under the scheme, NHI patients have to cover the costs themselves, purchasing the goods from private pharmacies. Further, under the Ministry of Health Instruction No. 0476 for the NHI, those who are categorised as poor, as certified by the district governor, should receive a food and transportation allowance from their health facility.

Implementation of the NHI began in 2016. Anecdotally, there have been some challenges in implementing the scheme, especially at the lower levels of the health system. The challenges relate in part to people’s awareness of their rights and healthcare staff understanding of the NHI. Other anecdotal challenges include the high cost of drugs where there are additional payments for medications not included under the NHI. The purpose of the current study was to assess: i) the percentage of patients recorded as NHI patients with co-payment, but who paid full or extra user fees based on individual billing payment; and ii) the factors related to overpayment.

## Methods

This is a descriptive cross-sectional study based on a structured exit questionnaire administered at the health facility level in face-to-face interviews with inpatients and outpatients. Outpatients or caregivers were randomly selected in front of the pharmacy unit at the health facility. All included inpatients or caregivers were also randomly selected and interviewed on exit. The team spent three days on average at each health facility. Eligibility criteria for participation were out- and in-patients regardless of age, diseases and with mild to moderate severity. Exclusion criteria related to those with severe or terminal illnesses.

### Study site

The geographical administrative units in Lao PDR consist of 17 provinces, 148 districts and 8,507 villages, with the village being the lowest administrative level [16]. In total, there are five central hospitals, 17 provincial hospitals, 143 district hospitals and 860 health centres [16]. The present study was conducted in the six provinces that began implementing the NHI in 2016 and have been implementing the NHI longer than other provinces, namely: Saravan, Attapeu, and Sekong in the southern part of the country; Borikhamxay in central part; Xieng Khouang and Luang Namtha in northern part of Lao PDR.

### Sample size and method

Multi-stage sampling was used, with the number of public health facilities within each province listed. Health facilities were then stratified by health centre (HC), district level healthcare (DH) facility and provincial health (PH) level. Based on this, cluster sampling was used to select one PH facility, two DH facilities and four HCs in each province (same catchment area of household surveys of the informal health insurers). Systematic random sampling was used for selecting outpatients and inpatients of each selected health facility for the exit interviews (4 HCs, 2DHs, 1PH).

The sample size for the exit interviews was 160 at each provincial level, 110 at the 2 district level and 40 at 4 health center level based on the statistical significant with 95% confidence level and 5% margin of error and the density of the service utilisation at each level. The estimated sample of patients at each province was 310 and the total sample for exit interviews was 1,860 patients. The sampling interval varied from every one to every three patients, depending on the patient volume reported by the facility manager.

### Data collection

Two teams of seven enumerators from the authors’ institutes and the Swiss Red Cross conducted the study. Data collectors were trained on the study protocol, instruments and guidelines in conducting interviews. The training went through each question in the questionnaire to ensure trainees understood the link between each question, as well as discussion of any possible confusing responses that may be encountered. All teams completed the initial pilot assessment in Borikhamxay province prior to the teams splitting into the remaining provinces, with each team collecting data from two provinces. Questionnaires were administered in face-to-face interviews. Further quality control, checking and cleaning was done in the office by the Lao Tropical and Public Health Institution (LaoTPHI) and University of Health Sciences (UHS) quality control team. The final, cleaned and consolidated dataset was created a few weeks after the completion of data collection.

The questionnaire included socio-demographic variables, living arrangements, distance from home to health facilities, type of health insurance, general patient payments at the health facility, payments by in-patients and out-patients for the referral and medical services, payments during hospitalisation, provision of NHI eligibility documents and knowing about NHI. The payments included direct medical costs for medicine/supplies, auxiliary tests, service charges, documents, referral fees, co-payments, purchasing medicines and supplies outside the hospitals and other payments.

Based on the type of health insurance and payments for each service, participants were categorised into four groups of payment type: 1) above the scheduled co-payment and other costs (e.g. medical supplies, auxiliary test, service charge, documents, co-payment, referral fee, accommodation, transport, incidentals); 2) scheduled co-payment; 3) OOP and 4) exempted payment for NHI/poor, free MCH services and free services for children under 5 years (see Table 2). For analysis, participants were further grouped into 2 categories of payment: 1) above the scheduled co-payment and other costs and OOP and 2) scheduled co-payment and exempted payment.

**Table 1.** Characteristics of 1,850 in-patient and out-patient respondents.

	In-patients				Out-patients					
	PH		DH/HC		PH		DH/HC		TOTAL	
Characteristic	N 143	%	N 102	%	N 471	%	N 1,134	%	N 1,850	%
**Age group**										
<1 yr	2	1.4	2	2	21	4.5	80	7.1	105	5.7
1 to 4 yr	13	9.1	18	17.6	61	13	232	20.5	324	17.5
5 to 17 yr	19	13.3	15	14.7	39	8.3	151	13.3	224	12.1
18 to 59 yr	90	62.9	52	51	285	60.5	594	52.4	1021	55.2
60 yr and over	19	13.3	15	14.7	65	13.8	77	6.8	176	9.5
**Sex**										
Male	98	68.5	55	53.9	284	60.3	671	59.2	1108	59.9
Female	45	31.5	47	46.1	187	39.7	463	40.8	742	40.1
**Occupation of patient**										
Self employed	5	3.5	0	0	23	4.9	26	2.3	54	2.9
Work for family (no salary)	48	33.6	23	22.5	148	31.4	294	25.9	513	27.7
Unemployed	13	9.1	3	2.9	65	13.8	76	6.7	157	8.5
Student	10	7	14	13.7	49	10.4	114	10.1	187	10.1
Other (Child, Housewife)	67	46.9	62	60.8	186	39.5	624	55	939	50.8
**Resident in this province or district**										
Yes	113	79	95	93.1	406	86.2	1,094	96.5	1708	92.3
No	30	21	7	6.9	65	13.8	40	3.5	142	7.7
**Distance from home to facility**										
Less than 1 km	4	2.8	4	3.9	12	2.5	168	14.8	188	10.2
1 to 5 km	29	20.3	36	35.3	130	27.6	442	39	637	34.4
6 to 10 km	23	16.1	13	12.7	69	14.6	171	15.1	276	14.9
11 to 30 km	31	21.7	26	25.5	111	23.6	189	16.7	357	19.3
Over 30 km	48	33.6	10	9.8	81	17.2	45	4	184	9.9
unknown	8	5.6	13	12.7	68	14.4	119	10.5	208	11.2
Asked to provide NHI eligibility documents										
Yes	115	80.4	88	86.3	344	73	740	65.3	1287	69.6
No	28	19.6	14	13.7	127	27	394	34.7	563	30.4
Showed documents										
Yes	125	87.4	93	91.2	408	86.6	935	82.5	1561	84.4
Did not bring document	10	7.0	5	4.9	33	7.0	140	12.3	188	10.2
Did not have document	8	5.6	4	3.9	30	6.4	59	5.2	101	5.5
Have heard about NHI										
Yes	77	53.8	52	51.0	273	58.0	532	46.9	934	50.5
No	66	46.2	50	49.0	198	42.0	604	53.3	918	49.6

**Table 2.** Types of payment at health facilities by 1,850 patients.

General patient payment at health facility	In-patient				Out-patient				Total	
	PH		DH/HC		PH		DH/HC			
	N 143	%	N 102	%	N 471	%	N 1,134	%	N 1,850	%
Co-payment only	40	28.0	45	44.1	214	45.4	516	45.5	815	44.1
Co-payment with other cost	14	9.8	5	4.9	23	4.9	48	4.2	90	4.9
OOP	24	16.8	12	11.8	109	23.1	158	13.9	303	16.4
Exempted payment	65	45.5	40	39.2	125	26.5	412	36.3	642	34.7
Total	143	100	102	100	471	100	1,134	100	1,850	100

### Statistical analysis

The statistical package, STATA was used to analyse the data. Frequency distributions of each variable of interest including socio-demographic characteristics of patients, type of health insurance among patients, payment of in-patients and out-patients were conducted. Descriptive statistics such as mean, median, frequency and percentage of the variables were analysed. Only variables with p < 0.025 in the univariate analyses were adjusted and included in the multiple logistic regression. Multiple logistic regression determined factors associated with the co-payments by using the backward stepwise method of elimination. Statistical significance was established at p < 0.05 and all tests were 2-tailed. Odds ratios and 95% confidence intervals were calculated.

### Ethical approval

Ethical clearance was given by the National Ethical committee for research, Ministry of Health, Lao PDR with the number 031, dated 12/03/2018. Verbal informed consent (approved by the Ethical Committee) was given by each respondent and each key informant prior to commencement of the interview. All documents were de-identified. Informed consent was obtained before conducting the interviews with caregivers or mothers who gave consent for children. Privacy and confidentiality were assured.

## Results

### Characteristic of inpatient and outpatients

Table 1 presents the characteristics of in- and out-patients. In total, 1,920 patients were selected for interview with 1,850 patients (96.3%) consenting to participate in the study. Two thirds of participants 59.9% of participants were male and 40.1% female. Of these, 61.2% of in-patients were male, while 59.8% of out-patients were female. Regarding age groups, 55.2% of respondents were aged 18–59 years old, and 17.5% were children under five years old. Most respondents (92.3%) lived in the same province or district where the facility they attended was located, with 86.1% of in-patients and 91.3% of out-patients living in the province or district. All participants worked in the informal sector, with the main occupation of participants (27.7%) being unpaid and working at home or other duties (28.1% in-patients and 28.7% out-patients); and 8.5% identified as being self-employed (6.0% in-patients and 10.2% out-patients). In total, 34.4% lived 1 to 5 km from the hospital, with 19.3% of respondents living 11 to 30 km away.

### Patients’ payments at health facilities

In total, 78.8% of respondents (77.9% of in-patients and 79.5% of out-patients) made co-payments or were exempted from payment. Among all NHI users, 44.1% were co-payment only and 34.7% were exempted from payment. For in-patients, high payments (co-payment with other costs (e.g. auxiliary tests, service charges, documents and OOP) were paid by 27.6% of provincial hospital and 16.7% of DH and HC patients, respectively). The total number of out-patients reporting additional payment to the health facility was approximately 28.0% and 18.1% at the PH level and DH/HC level, respectively (Table 2).

### Payments by in-patients and out-patients

Table 3 illustrates the payment of in-patients and out-patients for referral and medical services. Among those in-patients who paid for services, 66.9% paid for medical (non-surgical) services, 13.9% paid for normal deliveries and 13.5% for surgery. Among those referred to the health facilities (n = 23), 30.4% paid for referral services.

About 65.2% of NHI users (55.6% of in-patients and 56.7% of out-patients) paid for health services. The median payment of medical services for in-patients at the PH was higher than DH/HC (263,000 LAK vs 48,000 LAK), while the median payment for medical services for out-patients at PH and DH/HC was not substantially different (30,000 LAK vs 22,000 LAK). The median payment of auxiliary tests for in-patients at the PH was higher than DH/HC (65,000 LAK vs 84,000 LAK), while the median payment for medical services for out-patients at PH and DH/HC was not substantially different (40,000 LAK vs 50,000 LAK). The median payment of auxiliary tests for in-patients at the PH was higher than DH/HC (50,000 LAK vs 100,000 LAK). Among those paid, 48.5% had a separate receipt (39.4% and 60.6% of in-patients and out-patients, respectively).

### Payment during hospitalisation

Regarding payments made by in-patients during hospitalisation, 22.4% paid for transportation with the median of payment of 500,000 LAK. About one-fourth of participants (19.2%) paid for food during hospitalisation with the median payment of 150,000 LAK. About half (47.3%) paid for personnel costs during hospitalisation with the median of payment of 50,000 LAK.

Just under half of hospitalised patients (48.2%) were admitted to hospital for 2–3 days. In total, 2.4% received a food allowance, and among these, 47.4% did not receive a food allowance every day. None of the participants received a transportation allowance.

### Factors associated with the payments above the scheduled fees of in-patients and out-patients

Table 4 shows a multiple logistic regression analysis of factors associated with payments by in-patients and out-patients. Factors significant at the p < 0.025 in the univariate analysis were included in the multiple logistic variables: age of patient, gender of patient, type of facility, patient occupation, whether the patient lived in the respective province or district, distance from their residence to the health facilities, ward services (in-patient non-surgery), referral, purchasing medicine or equipment outside the health facilities, being asked to provide NHI eligibility documents, showing eligibility documents voluntarily. Significant factors associated with in-patients paying above the scheduled fee were living in the province and district (OR = 2.8; 95%CI 1.2 to 6.3); not having documents with them (OR = 21.2; 95%CI 5.6 to 80.3); or not having documents (OR: 7.8; 95% CI 2.1 to 28.6). Significant factors associated with additional costs for out to patients were level of facility used at the provincial hospital (OR:1.4; 95% CI 1.1 to 1.9); older age (OR = 2.2; 95%CI 1.5 to 3.1); living in the province and district (OR = 2.3; 95%CI 1.5 to 3.7); living more than 5 km from the facility (OR = 1.4; 95%CI 1.1 to 1.9); buying medicine or supplies outside of the health facility (OR: 5.6; 95% CI 3.1 to 10.2); not bringing documents (OR:9.1; 95% CI 6.1 to 13.5), not having the right documents (OR: 8.9; 95% CI 5.4 to 14.8).

**Table 3.** Payment of in-patients and out-patients for referral and medical services.

	In-patients				Out-patients					
	PH		DH/HC		PH		DH/HC		Total	
NIH users	N 143	%	N 102	%	N 471	%	N 1134	%	N 1850	%
Ward of service										
In-patient (non-surgery)	80	55.9	84	82.4					164	66.9
Surgery	28	19.6	5	4.9					33	13.5
Normal delivery	25	17.5	9	8.8					34	13.9
Caesarean	2	1.4	1	1.0	Outpatient data not available				3	1.2
Miscarriage	8	5.6	3	2.9					11	4.5
Referred										
Yes	17	11.9	1	1.0					23	8.0
No	126	88.1	101	99.0					266	92.0
Pay for the referred										
Yes	6	35.3	0	0.0					7	30.4
No, not paid	10	58.8	1	100					15	65.2
DK	1	5.9	0	0.0					1	4.3
Payment at the facility										
Yes	80	52.8	62	58.4	344	55.1	721	58.3	1207	65.2
No	63	47.2	40	41.6	123	44.3	407	41.2	633	34.2
DK	0	0	0	0	4	0.6	6	0.5	10	0.5
Payments for medical/supplies (in 1000 LAK)										
Median	263		48		30		22			
Min	20		26		1		3			
Max	1460		1050		230		120			
Payments for auxiliary tests* (in 1000 LAK)										
Median	65		84		40		50			
Min	20		10		4		20			
Max	1040		130		225		140			
Payment for service charge (in 1000 LAK)										
Median	25		25		15		15			
Min	25		20		5		8			
Max	25		30		70		80			
Payment for documents (in 1000 LAK)										
Median	20		20		10		15			
Min	15		10		5		5			
Max	50		30		30		40			
Payment for co-payment (in 1000 LAK)										
Median	30		30		15		15			
Min	10		10		15		10			
Max	30		35		30		35			
Payment for referral fee (in 1000 LAK)										
Median	20		22.5		-		-			
Min	5		20		-		-			
Max	300		25		-		-			
Payment for bed/room (in 1000 LAK)										
Median	100		50		-		-			
Min	5		30		-		-			
Max	525		180		-		-			
Paid for co-payment schedule										
Yes, separate receipt	36	45.0	21	33.9	203	86.4	196	34.8	456	48.5
Yes, include with other receipt	2	2.5	6	9.7	1	0.4	29	5.2	38	4.0
Yes, but no receipt	13	16.3	21	33.9	31	13.2	331	58.8	396	42.1
No, paid different amount	2	2.5	3	4.8	0	0.0	7	1.2	12	1.3
No answer	27	33.8	11	17.7	0	0.0	0	0.0	38	4.0
Buying medicines or supplies outside health facility										
Yes	3	2.1	5	4.9	33	7.0	29	2.6	70	3.8
No	140	97.9	97	95.1	438	93.0	1,105	97.4	1780	96.2

*Auxiliary tests included CBC, Biochemistry tests …

**Table 4.** Multiple regression analysis factors associated with payments exceeding scheduled fees for in-patients and out-patients.

Factor	In-patients (N = 55)						Out-patients (N = 338)					
	Payment		Crude		Adjusted		Payment		Crude		Adjusted	
	N (55)	%	OR	95%CI	AOR	95%CI	N (338)	%	OR	95%CI	OR	95% CI
Facility use												
DH/HC	17	16.7	1				206	18.2	1		1	
PH	38	26.6	1.9	1.0 to 3.4			132	28	1.8	1.4 to 2.3	1.4	1.1 to 1.9
Are you a patient?												
Yes	29	22.8	1				208	23.5	1			
No	26	22	1	0.5 to 1.7			130	18.1	0.7	0.6 to 0.9		
Age of patient												
Under 5 yr	5	14.3	1				54	13.7	1		1	
5–17 yr	6	17.7	1.3	0.4 to 4.7			36	19	1.5	0.9 to 2.3	1.7	1.1 to 2.9
18 yr and over	44	25	2	0.7 to 5.5			248	24.3	2	1.5 to 2.8	2.2	1.5 to 3.1
Gender of patient												
Female	35	22.9	1				200	20.9	1			
Male	20	21.7	0.9	0.5 to 1.7			138	21.2	1	0.8 to 1.3		
Occupation of patient												
Private/state enterprise/Self-employed/student	7	24.1	1				55	25.9	1			
Unemployed	23	26.4	1.1	0.4 to 3.0			127	21.8	0.8	0.6 to 1.1		
Other	25	19.4	0.8	0.3 to 2.0			156	19.3	0.7	0.5 to 1.0		
Patient from this province/district												
Yes	39	18.8	1		1		294	19.6	1		1	
No	16	43.2	3.3	1.6 to 6.9	2.8	1.2 to 6.3	44	41.9	3.0	2.0 to 4.4	2.3	1.5 to 3.7
Distance from home to facility												
≤5 km	14	19.2	1				137	16.5	1		1	
>5 km	41	23.8	1.3	0.7 to 2.6			201	21.3	1.4	1.1 to 1.8	1.4	1.1 to 1.9
Ward of service												
Medical (non-surgery)	30	18.3	1									
Surgery/delivery/miscarriage	25	30.9	2	1.1 to 3.7								
Referred												
Yes	2	11.1	1									
No	53	23.3	2.4	0.5 to 10.9								
Buy medicine or supplies outside this health facility												
No	51	21.5	1				302	19.6	1		1	
Yes	4	50	3.6	0.8 to 15.0			36	58.1	5.7	3.4 to 9.6	5.6	3.1 to 10.2
Asking to provide NHI eligibility documents												
No	11	26.2	1				80	15.4	1		1	
Yes	44	21.7	0.8	0.4 to 1.7			258	23.8	1.7	1.3 to 2.3	3.0	2.2 to 4.2
Showed documents												
Yes	35	16.1	1		1		206	15.3	1		1	
No, didn’t bring document	12	80	18.7	5.1 to 68.9	21.2	5.6 to 80.3	85	49.1	5.3	3.8 to 7.4	9.1	6.1 to 13.5
No, didn’t have document	8	66.7	10.5	3.1 to 35.6	7.8	2.1 to 28.6	47	52.8	6.2	4.0 to 9.6	8.9	5.4 to 14.8
Paid for transportation, food, personal costs during hospitalisation												
No	4	16.7	1									
Yes, at least 1 item	51	23.1	1.5	0.5 to 4.6								
Number of days admitted												
1–3 days	39	21.3	1									
≥4 days	16	25.8	1.3	0.7 to 2.5								
Heard about NIH												
Yes	26	20.2	1				173	21.5	1			
No	29	25	1.3	0.7 to 2.4			265	20.6	0.9	0.7 to 1.2		

## Discussion

Increasing global attention is being given to UHC as highlighted in the Sustainable Development Goals. Within this context, many countries without universal access are undertaking health financing reform to ensure households are protected from high OOP and are able to access the health services they need in a timely manner [2,8,17–21]. As in other countries in the region such as Thailand and Vietnam [22,23], Lao PDR, in its efforts to achieve UHC, is shifting to a single-coverage NHI program as opposed to having multiple schemes for different sub-populations [8]. As implementation of the scheme is in its early stages, it is too early to fully evaluate the effect of the NHI. Nevertheless, we aimed to examine several points: whether or not patients are making additional co-payments; what determines whether they make co-payments above the scheduled fee; and what percentage of patients recorded as NHI patients paid too much due to co-payment, or paid full or extra user fees based on individual billing payment.

In this study, a total, 11.9%, of 1,850 respondents were NHI general patients and paid the correct co-payment at the health facility. The study revealed that about 20% paid above the defined amount of payment for out- and in-patient services. These additional expenditures may be due to having to buy medicines or supplies not available in the public healthcare facilities or not covered by the scheme. If this is the case, it is possible providers direct insured patients to services, drugs, and tests not covered by the insurance scheme as a means of generating more revenue. It is also possible service providers charge additional co-payments that are higher than the government reimbursements, with just under half having a separate receipt. It may also be the case that patients choose to pay extra for a faster or higher quality service. Of note, however, is that the OOP payment is the net effect of change in unit price and amount of services consumed, and OOP expenditure is not necessarily negative [2]. It may be those within the scheme are willing or more able to pay for additional services and tests that would not be available in the absence of insurance [2].

Other reasons for paying more than scheduled fees could include rising unit costs of health services, increased patient charges and the negative NHI impact on healthcare consumption, resulting in a rise in total health spending for the patients. None of the eligible patients received subsidies for indirect costs such as transportation and meals during hospitalisation, and those who did receive the meal allowance did not receive it every day. Not receiving the travel allowance probably explains why living more than 5 km from the facility was associated with co-payments. Additional costs on top of the scheduled co-payment and not receiving reimbursement for food and travel can be an important deterrent for seeking care for those living in remote areas, who are generally also amongst the poorest accessing healthcare [24–27].

While the number of social health protection coverage (formal workers and informal population) is over 90%, over two-thirds of participants did not know which scheme they were enrolled in and did not bring, nor have, relevant, necessary documents about contributing to co-payments. This suggests poor knowledge of the scheme and how it works. Other studies demonstrate a low level of awareness of health insurance in developing countries especially among the informal sector [28–30]. Sensitisation to, and knowledge about, an issue is usually needed to raise interest, understanding and active participation [31,32]. The patients without documents may not have yet been issued certificates as evidence for accessing health services at public health facilities, or individuals who have migrated from one province to another may not yet have a family book, usually issued by chiefs of villages.

While we did not look at population level enrolment, within the study sample 14.5% did not think they belonged to any insurance scheme. This figure is likely to be lower than the actual percentage of people not belonging (or understanding they belong to the scheme) as we only interviewed patients. While the NHI scheme is tax-based system for the informal workforce with no formal enrolment into the NHI required as individuals are eligible when they use healthcare services based on the NHI rules, it is possible not all eligible individuals are aware of this. This may be especially the case for informal workers with low levels of literacy. This also highlights the need for increasing awareness of the scheme amongst individuals and providers, as has been observed elsewhere in lower-middle income countries in the early stages of NHI scheme implementation [17,20].

The study did not examine quality of care, which nevertheless remains a major challenge for Lao PDR despite steps towards improvement [21,33–35]. Quality of care however is of vital importance to the performance of health insurance schemes and enrolment. For example, in Lao PDR, one study found the third most important reason for civil servants not enrolling in the earlier civil servants’ scheme was poor quality of government hospitals [21]. A study of an earlier voluntary community-based health insurance targeting for the informal sector and self-employed workers was also reported to be low due to poor quality of services [29]. It is possible therefore substantial uptake in insurance also requires improvements in the quality of care provided by healthcare services.

### Limitations of the study

We acknowledge that the findings of the study cannot be generalised beyond the six included provinces and twelve district offices of each the NHI scheme in each of the Southern, Central and Northern regions of Lao PDR. Further, the study covered the implementation process from 2018 to 2019, but it is acknowledged that policy and implementation processes are complex and can be affected by both endogenous and exogenous factors, which were not captured in this study. Nor did we interview people who may have needed healthcare but did not seek care. Limitations notwithstanding, this study contributes to the evaluation of the Lao PDR’s NHI implementation in particular, and evaluation of health insurance in general.

## Conclusion

Attaining universal population coverage for health insurance in other countries has taken several decades and it is not surprising some people did not know details about the insurance scheme in which they were enrolled, and if it existed at all. Some patients also paid more than required, while other eligible patients did not receive the food allowance and transport allowance for round trip home. This may be due to lack of knowledge or not feeling able to challenge healthcare providers. This suggests the need for increased awareness raising and engagement with both NHI enrolees and healthcare staff. More research is needed to determine the factors that contributed to co-payments and lack of reimbursement for travel and food as these additional costs may act as a deterrent for some people in accessing healthcare.

## Paper context

In many lower-middle income countries a large proportion of the workforce is in the informal sector with no health insurance. In Lao PDR to address this, the government is implementing national insurance schemes. In this study, we found not all participants were aware of the scheme, some paid more than they should have while others did not receive all the benefits they should have.
